# A Brain-Inspired Homeostatic Neuron Based on Phase-Change Memories for Efficient Neuromorphic Computing

**DOI:** 10.3389/fnins.2021.709053

**Published:** 2021-08-19

**Authors:** Irene Muñoz-Martin, Stefano Bianchi, Shahin Hashemkhani, Giacomo Pedretti, Octavian Melnic, Daniele Ielmini

**Affiliations:** Dipartimento di Elettronica, Informazione e Bioingegneria (DEIB), Politecnico di Milano, Milan, Italy

**Keywords:** brain-inspired computing, unsupervised learning, reinforcement learning, spike-timing-dependent plasticity, hardware resilience, homeostatic scaling, synaptic scaling, phase change memory

## Abstract

One of the main goals of neuromorphic computing is the implementation and design of systems capable of dynamic evolution with respect to their own experience. In biology, synaptic scaling is the homeostatic mechanism which controls the frequency of neural spikes within stable boundaries for improved learning activity. To introduce such control mechanism in a hardware spiking neural network (SNN), we present here a novel artificial neuron based on phase change memory (PCM) devices capable of internal regulation via homeostatic and plastic phenomena. We experimentally show that this mechanism increases the robustness of the system thus optimizing the multi-pattern learning under spike-timing-dependent plasticity (STDP). It also improves the continual learning capability of hybrid supervised-unsupervised convolutional neural networks (CNNs), in terms of both resilience and accuracy. Furthermore, the use of neurons capable of self-regulating their fire responsivity as a function of the PCM internal state enables the design of dynamic networks. In this scenario, we propose to use the PCM-based neurons to design bio-inspired recurrent networks for autonomous decision making in navigation tasks. The agent relies on neuronal spike-frequency adaptation (SFA) to explore the environment via penalties and rewards. Finally, we show that the conductance drift of the PCM devices, contrarily to the applications in neural network accelerators, can improve the overall energy efficiency of neuromorphic computing by implementing bio-plausible active forgetting.

## 1. Introduction

The field of artificial intelligence (AI) has recently seen significant breakthroughs in the research, showing high performance in several tasks such as image recognition, natural language processing and playing games (Collobert et al., 2011; Krizhevsky et al., 2012; Mikolov et al., 2012; Silver et al., 2016). The most widespread approach to AI has focused on deep learning, where the intelligent systems are trained via specific algorithms such as backpropagation (LeCun et al., 2015). However, the pre-tuning of the training parameters, which requires time and power intensive procedures, deprives the systems of the plastic adaptation to the environment which, on the other hand, is one of the fundamental properties of the biological organisms. This lack of resilience with respect to a constantly changing environment is what actually hinders the current AI to achieve human-like accuracy in daily-life tasks (Parisi et al., 2019).

Biological organisms collect, settle and modulate the information relying on specific mechanisms of synaptic plasticity and neural activity (Turrigiano, 1999). In particular, the learning procedure is usually explained in terms of Hebbian-type plasticity, where the time correlation between the pre-synaptic and post-synaptic spikes induces variations of the synaptic weights (Fox and Stryker, 2017; Lisman, 2017), as in spike-timing-dependent plasticity (STDP) (Masquelier and Thorpe, 2007). On the other hand, Hebbian learning cannot completely describe the learning procedure of the brain, since the only STDP theory foresees a continual synaptic potentiation and depression as a consequence of the correlation between the neuronal responses and the corresponding inputs (Miller and MacKay, 2008). In fact, biological systems adopt homeostatic regulation to keep the overall neuronal and synaptic activities within safe boundaries, which also helps to counteract unwanted changes of the firing rate due to external perturbations (Turrigiano, 1999). In this framework, the synaptic scaling, or homeostatic scaling (Turrigiano, 2008), refers to the biological mechanism able to counteract a chronically high firing rate of a population of neurons. Thus, Hebbian learning and homeostatic regulation sustain each other for the optimization of experience-based knowledge toward continual adaptation of real-life information (Abraham and Robins, 2005; Zenke et al., 2017).

Experience-based knowledge, where agents learn a behavioral policy by interacting with the world and consequently receiving penalties and rewards, is a scientific field shared between neuroscience and computer science known as “reinforcement learning” (Kaelbling et al., 1996). One of the leading reinforcement mechanism is associated with dopamine, a pleasure-related neurotransmitter, which is released in the brain when a person succeeds in solving a problem (Schultz et al., 1997). In the literature, several approaches have been proposed to facilitate reinforcement learning. For instance, reinforcement techniques have been shown to enable the learning of optimized behavioral policy for a given model of the space, where the agent continually looks for the maximization of the reward thus acquiring an accurate mapping of the environment (Sutton, 1988). However, in real life, an agent must build its own model by incremental experience of positive and negative events, as studied by model-free methods such as (i) Q-learning (Watkins and Dayan, 1992) and (ii) temporal difference learning, TD(λ) (Doya, 2000). In particular, in the last few years, such cognitive functions have been widely discussed in the framework of attractor neural networks for the key role of cognitive functions, such as context dependent decision making (Doya, 2000; Kuzum et al., 2012), thus gaining momentum as viable networks to replicate human-like behaviors (Chicca et al., 2014).

The combination of the benefits introduced by homeostatic mechanism and reinforcement learning would thus improve the artificial intelligence systems toward the ability to autonomously interact with the environment in real life situations.

In this framework, several neuromorphic spiking neural networks (SNNs) based on CMOS technology have been proposed, demonstrating VLSI synaptic circuits with homeostatic neurons (Bartolozzi and Indiveri, 2006; Chicca et al., 2014; Qiao et al., 2017) and reward-based decision-making circuits (Wunderlich et al., 2019; Yan et al., 2019). At the same time, non-volatile memory devices, such as phase change memory (PCM), have raised considerable interest as promising synaptic connections for neuromorphic computation, thanks to the 3D stacking capability, the low-voltage operation and the ability to serve as embedded non-volatile memory in computing systems (Suri et al., 2012; Xu et al., 2020; Ren et al., 2021). In particular, PCMs have recently demonstrated outstanding multi-level capability (Kuzum et al., 2013; Ren et al., 2021), which enables continual learning in neural networks (Bianchi et al., 2019; Muñoz-Martín et al., 2019) and decision making in brain-inspired cognitive systems (Eryilmaz et al., 2014).

In this work, we present a novel artificial integrate-and-fire (I&F) neuron based on PCM devices implementing homeostatic mechanisms. In particular, the gradual crystallization of a PCM device enables the continual tuning of the internal threshold of the neuron as a function of the level of firing excitation. This adaptation process improves the learning capability and directly translates in hardware the homeostatic control mechanism that manages the synaptic weight update during STDP. We show that the homeostatic neuron can optimize the pattern specialization of large images, e.g., those taken from the Fashion-MNIST dataset, while enabling high robustness against errors and external perturbations (Muñoz-Martín et al., 2020). In this framework, we propose the use of PCM-based homeostatic neurons for achieving continual learning in standard convolutional neural network. We also analyze the impact of device programming failure in relation to the multilevel capability of the PCM devices. The impact of PCM conductance drift is also studied (Suri et al., 2012; Xu et al., 2020; Ren et al., 2021), demonstrating that this device non-ideality could implement bio-inspired features, such as active forgetting. Finally, we propose a novel bio-inspired recurrent neural network (RNN) capable of solving reinforcement learning tasks. The internal state of each neuron of the RNN is mapped by the self-adaptive threshold using a PCM device, which modulates, as before, the firing excitability. The more the neuron fires, the more the control PCM conductance increases, thus mapping the dynamic behavior of the network in real time (Bianchi et al., 2020b). In this work, the recurrent PCM device enables the study of several reinforcement learning tasks such as decision making during autonomous navigation, with particular attention in terms of power-efficiency. This work highlights the importance of PCM devices as key elements to achieve adaptation, learning and autonomous navigation exploiting the benefits of local edge computing.

## 2. Bio-Inspired Learning in Artificial Neural Networks

**Figure 1:**
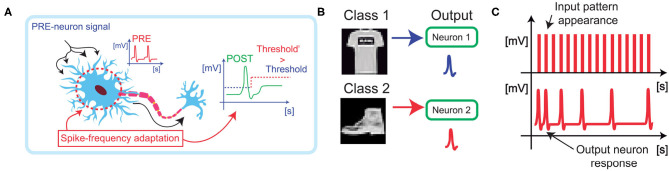
**(A)** Biological neurons are stimulated by spikes coming from the synaptic connections and modulate their response in frequency as a function of the spiking activity. **(B)** By implementing the spike frequency adaptation in hardware, it is possible to introduce a boundary for the learning activity of large images, e.g., from the Fashion-MNIST dataset, thus boosting the overall specialization accuracy. **(C)** Furthermore, the specialization of the output neuron results in a decrease the firing activity of the neuron, thus optimizing the energy consumption.

Figure 1A shows a schematic illustration of spike-frequency adaptation (SFA) in a neuronal cell. When a signal excites a neuron, the output firing rate is balanced between an increase due to the synaptic potentiation and a decrease due to the homeostatic mechanism (Indiveri et al., 2011). In synaptic learning processes, this threshold regulation aims at stabilizing the learning activity and limiting the growth of the synaptic weights, thus enabling low energy consumption and better accuracy of classification.

The homeostatic adaptation has been studied in the case of a winner-take-all (WTA) network for the classification of large images. The output homeostatic neurons (POSTs) must specialize on different classes of images presented at the input of the WTA, Figure 1B, thus enabling the spike-frequency adaptive mechanism that limits the power consumption and enables efficient classification (Figure 1C; Pedretti et al., 2018). Classification is achieved by using both excitatory synapses, which evolve by increasing or decreasing the conductance accordingly to STDP, and inhibitory synapses, which prevent the same specialization on different patterns by discharging the integration at each POST firing activity (Bianchi et al., 2020a). Synaptic excitatory dynamics are reproduced by using PCM devices switching from low resistive state (LRS) to high resistive state (HRS), and vice versa. Potentiation is achieved when the POST fires after the pre-neurons (PREs), while depression is achieved when the POST fires before the PRE (Bianchi et al., 2020c).

### 2.1. Hardware Realization of the Homeostatic Neuron

**Figure 2:**
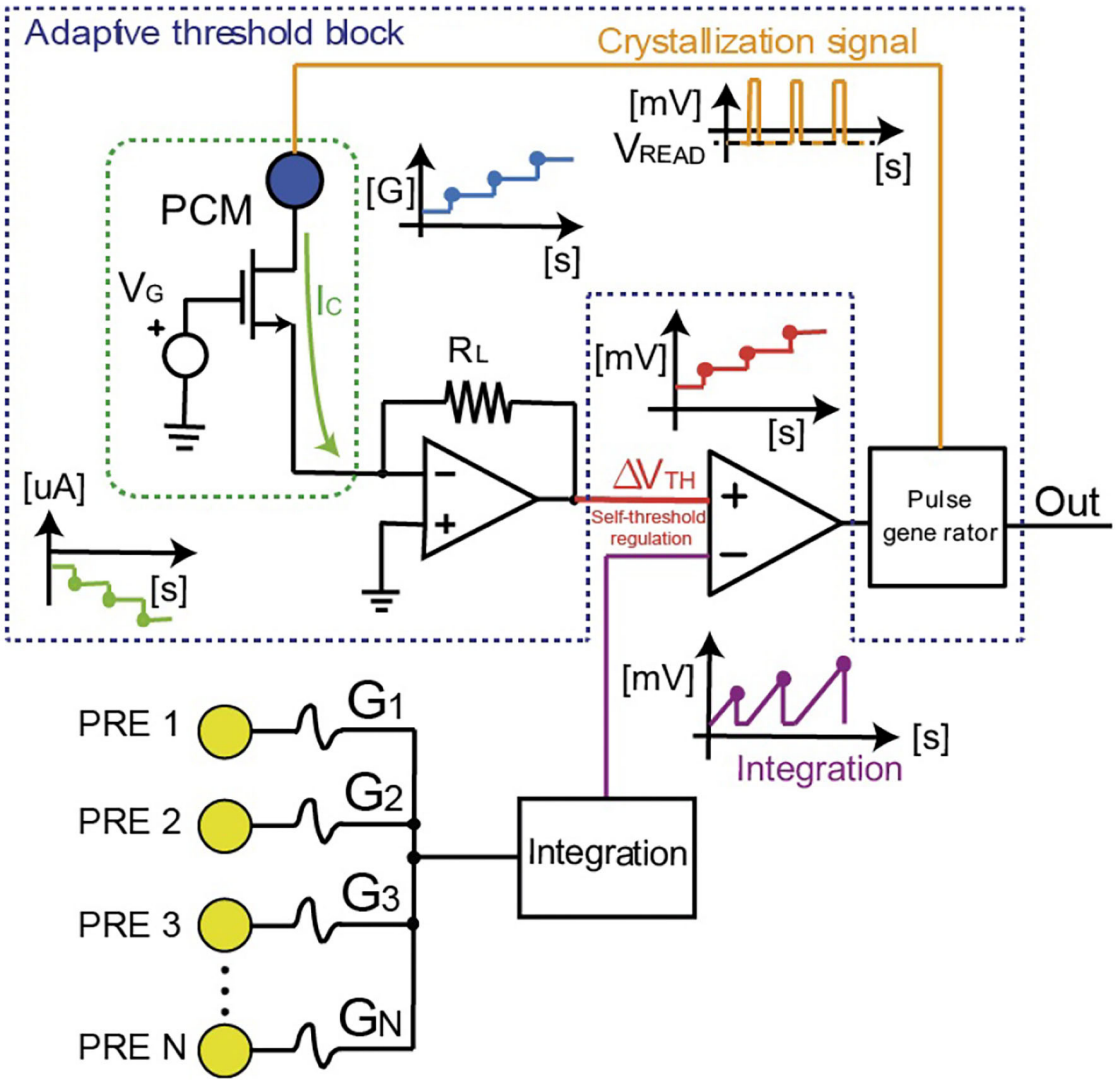
Scheme of the homeostatic neuron with the control PCM device which regulates the internal threshold. The spike signals coming from other neurons (e.g., pre-synaptic neurons) are integrated (“Integration” block) using an Arduino microcontroller (2 or Mega2560 in the measurements we performed). Arduino also manages the fire activity when the threshold of the neuron is overcome. When this happens, two signals are generated: (i) the “Out” response of the neuron and (ii) the crystallization pulse for the gradual increase of the PCM conductance. In this way the internal threshold *V*_*TH*_ of the neuron increases.

Figure 2 illustrates the artificial neuron circuit, where the threshold is managed by a control PCM directly connected to the comparator which compares the membrane potential with the threshold. PCM devices typically show multilevel storage with a large number of analog conductance states (Kim et al., 2019). In Figure 2, the multilevel behavior is obtained by the applications of repeated set pulses to the top electrode for gradual crystallization or amorphization, thus causing a modulation of the neuronal threshold (Suri et al., 2011; Wright et al., 2013; Tuma et al., 2016).

The incoming PRE spikes are weighted by PCM synapses which induce a synaptic current collected by the “integration” block in Figure 2. The synaptic current spikes are integrated until the internal potential hits the threshold of the neuron. This event causes the generation of two spikes, namely (i) a POST spike which is applied to the next layer of neurons, and (ii) a second spike which is applied to the top electrode of the internal PCM device to induce partial crystallization, which is responsible for a self-threshold regulation. Each crystallization pulse leads to an incremental set transition of the PCM device to higher conductive values G_*PCM*_. The PCM conductance is the leading element setting the responsivity of the neurons since it maps the fire threshold V_*TH*_ of the neuron. In particular, V_*TH*_ is obtained as the read current of the PCM biased at negative values (V_*read*_ < 0) after conversion by the trans-impedance amplifier of Figure 2, namely V_*TH*_ = −R_*L*_G_*PCM*_V_*read*_, where R_*L*_ is the feedback resistance and G_*PCM*_ also includes the conductance of the series transistor M_1_. Initially, the PCM device is prepared in the HRS, thus resulting in low current I_*C*_ and low threshold voltage V_*TH*_. As the POST fires, the incremental crystallization of the PCM causes the increase of the threshold with respect to the first reference firing value. The gradual crystallization procedure is thus iterated at every POST fire, causing a continuous increase of V_*TH*_. As a result, more input spikes are needed to induce the fire of the neuron or, equivalently, the spiking frequency of the POST decreases at increasing crystallization of the control PCM.

### 2.2. Characteristics of the PCM Devices

**Figure 3:**
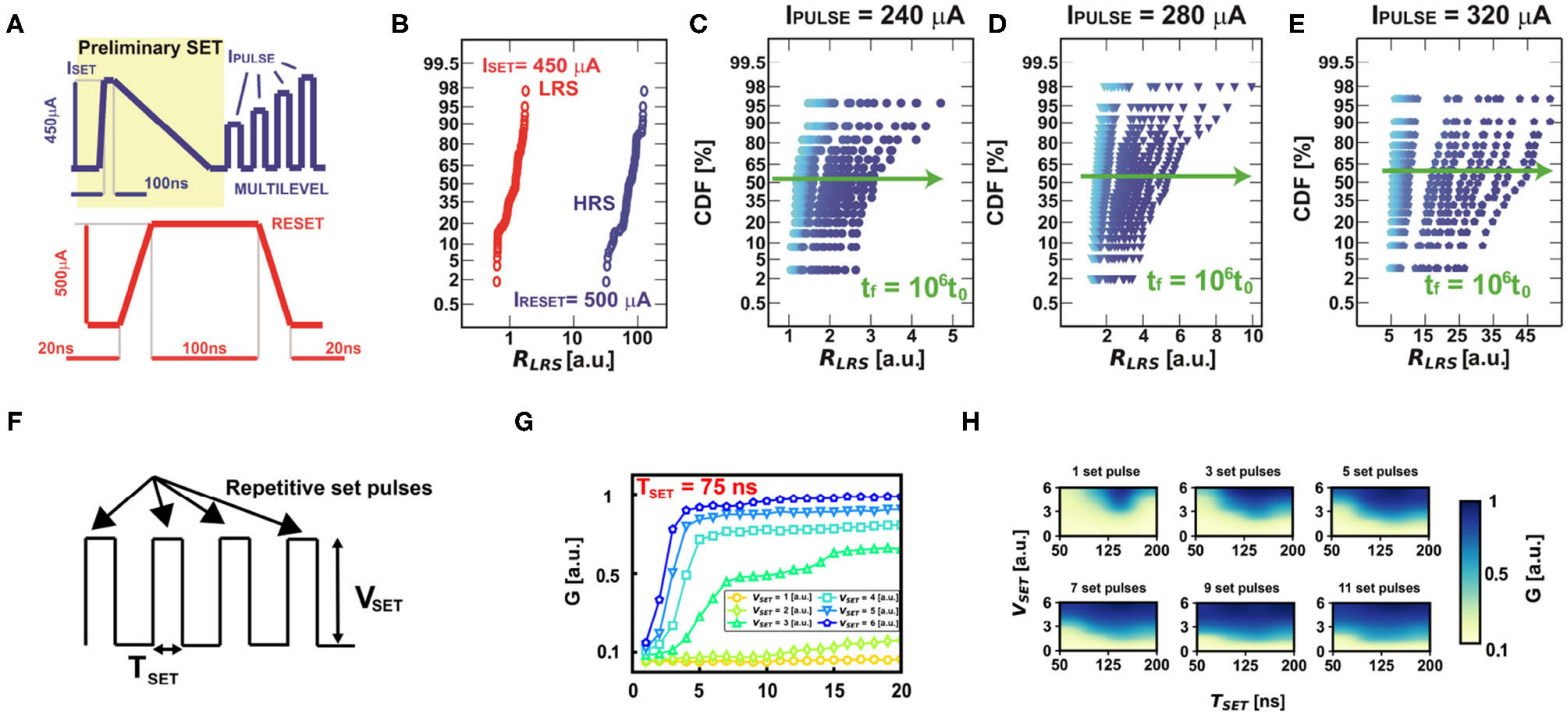
**(A)** Typical current programming signals used to set and reset the PCM device to low resistive state (LRS) and high resistive state (HRS), respectively. **(B)** LRS and HRS experimental distributions of the PCM devices. **(C–E)** Distributions of three different programmed multilevel resistive states to highlight the effect of the conductance drift in time. Note that the conductance drift is more evident at higher initial values of resistances. **(F)** Voltage-based rectangular programming pulses for achieving multilevel resistive states starting from a partial HRS. **(G)** Multilevel characteristics at different set voltages as a function of the number of rectangular pulses for a pulse duration T_*SET*_ = 75 ns. **(H)** Experimental color maps varying the number of pulses for achieving multilevel states as a function of the rectangular pulse amplitude and duration.

The PCM is programmed by set (with current I_*SET*_) and reset signal pulses as shown in Figure 3A. Figure 3B shows the cumulative distribution of the LRS and HRS resistances after the application of the programming signals, with two orders of magnitude of resistive window. On the other hand, note that the PCM shows a gradual increase of conductance which suitably reproduces the adaptive threshold regulation of V_*TH*_. In particular, the variation of LRS distributions can be modulated by proper choice of I_*PULSE*_, thus enabling multilevel states. The multilevel behavior of the PCMs can be obtained by both starting from a full LRS and applying incremental amorphizing pulses, as indicated in Figure 3A, or from a partial HRS and applying crystallizing pulses. Note that the crystallization depends on both the amplitude and duration of the pulses. In general, G_*PCM*_ is more easily modulated by using shorter pulses and intermediate set voltages. In this way, the conductive multilevel states can be spread over one order of magnitude, thus enabling the possibility of effective modulation of the threshold (Wong et al., 2010).

Note that the PCM resistance suffers from the conductance drift in time, which is due to the structural relaxation of the device (Kim et al., 2019). Figures 3C–E illustrate the time evolution of three different resistance distributions. Experimental data show that the conductance drift is higher for higher initial resistances, thus obtaining a non-linear increase in time of the initial programmed conductive value if the device is not continuously re-programmed. Such variation in time of the synaptic weights implemented with PCM devices is a key limitation for the design of neural accelerators (Kim et al., 2019; Joshi et al., 2020). The progressive decrease of the conductance also affects the homeostatic mechanism. However, the drift can also have a beneficial effect in our bio-inspired neuron, since it gives the possibility of spontaneous forgetting. In fact, the threshold of the neuron naturally decreases during drift, thus increasing the neuronal firing excitability and enabling an active forgetting mechanism.

Note that the PCM devices can be also programmed in multilevel states by applying repetitive voltage rectangular pulses, as highlighted in Figure 3F starting from a partial HRS. In particular, it is possible to modulate the number of multilevel states by proper choice of the voltage amplitude V_*SET*_ at fixed pulse duration T_*SET*_, as highlighted in Figure 3G for T_*SET*_ = 75 ns. Note also that it is possible to have a modulation of the resistive states at various combination of duration and amplitude of the repetitive programming pulses, as depicted in Figure 3H, thus giving rise to an extensive resistive modulation as a function of the target programming condition. This is very important for the development of neuromorphic and neural networks with PCM-based homeostatic neurons, as it is going to be analyzed in the following.

## 3. Unsupervised STDP With Homeostatic Mechanism

**Figure 4:**
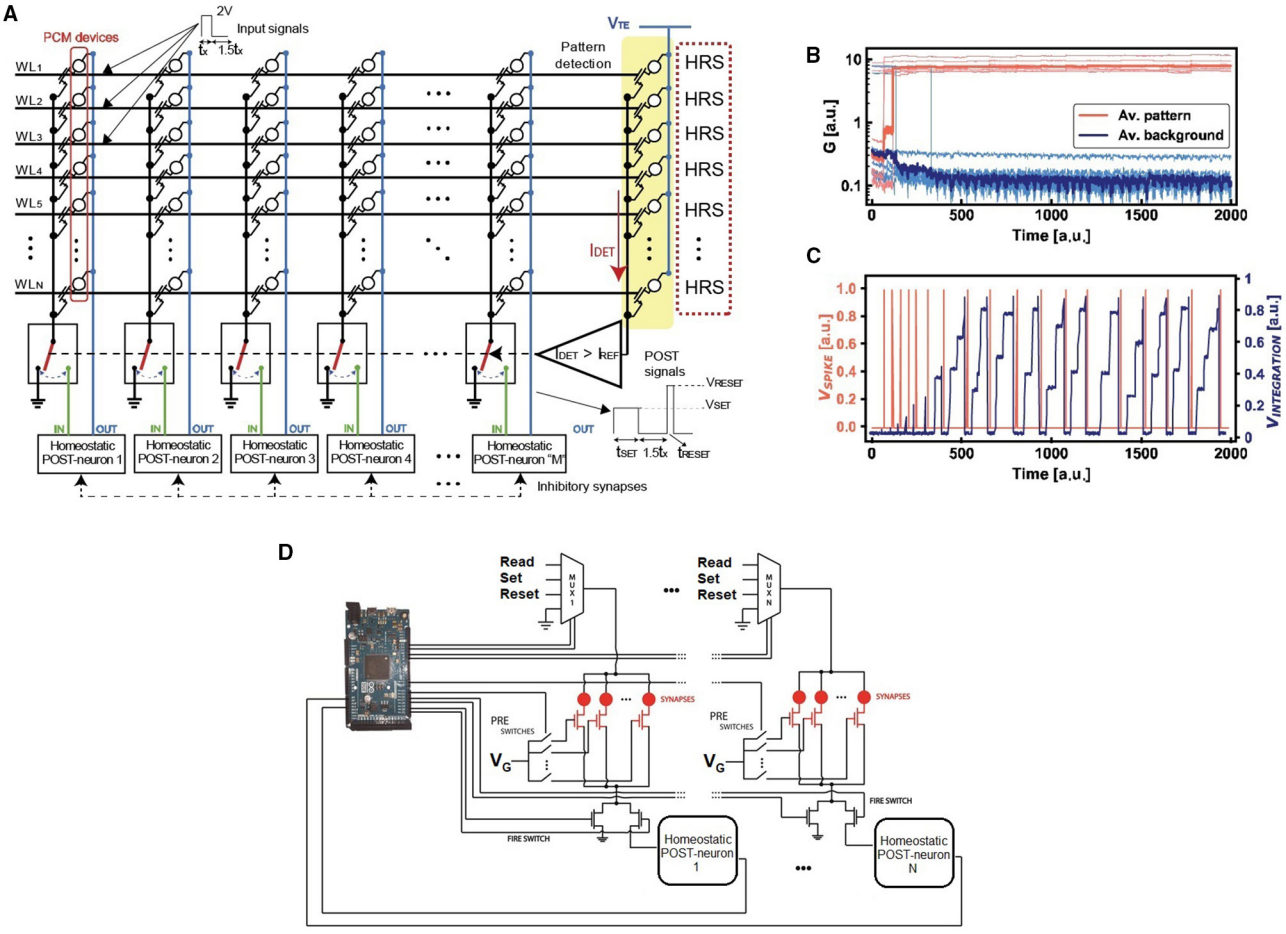
**(A)** Experimental setup for the asynchronous STDP, where the inputs are submitted at the wordlines (WLs). Every column connects the WLs to a specific POST by using 1T1R PCM excitatory synapses, while the inhibitory synapses are implemented via discharge signals of the integrators. A further column of HRS devices is used for pattern/noise detection. The integration activity of each neuron is enabled only for I_*DET*_ > *I*_*REF*_. **(B)** Experimental evolution of the pattern and background synapses under STDP. Note that the inputs are presented to the network asynchronously, since the potentiation and depression are gradual. **(C)** At every firing activity, the internal PCM device of the neuron is incrementally set thus obtaining an overall reduction of the spiking frequency due to the increase of the internal threshold of the neuron. This spike frequency adaptation enables optimized pattern specialization and reduced energy consumption. **(D)** Schematic representation of the experimental setup with several POST-synaptic neurons in order to implement a WTA network. Note that the microcontroller (we used both Arduino 2 and Arduino Mega2560) acts as master of the system.

To study the properties of the homeostatic neuron with respect to the classification accuracy of input images, we designed a spiking neural network capable of unsupervised learning by STDP. The input patterns are submitted asynchronously, which means that not all the patterns are presented with fixed density and shape to the network. Note also that the input signal consists of an alternation of the asynchronous pattern and random noise spikes, where noise, used for background depression, has lower density and input appearance probability in order to assure circuital and learning stability during operation (Bianchi et al., 2020c). Figure 4A illustrates the SNN, where PCM synapses have 1-transistor/1-resistor (1T1R) structure with the gates of the transistors connected by wordlines (WLs) and the PCM top electrodes connected by bitlines (BLs). The bitlines are directly linked to the neurons, since the feedback neuronal signal is used to adjust the synaptic weights involved in the STDP protocol (Ambrogio et al., 2016a). Thus, with respect to Figure 2, which represents the main structure of the homeostatic neuron, a further signal line is needed for the unsupervised learning with STDP. Input spikes are applied to the WLs to induce synaptic currents that are summed at each column to feed the I&F POSTs with self-adaptive threshold, according to the scheme of Figure 2. The feedback spike consists of a set pulse of voltage *V*_*TE*_, followed by a pulse of reset voltage. The overlap between the PRE spike and the POST spike induces potentiation (set transition) or depression (reset transition) for positive or negative delay between the two spikes (Bianchi et al., 2020c). During potentiation the synaptic element switches to LRS, while during depression the synaptic element switches to HRS. Thus, the STDP is mapped in a binary framework, which enables simpler hardware computation with respect to bio-inspired analog STDP (Bianchi et al., 2020c). Note that an extra column of PCM synapses programmed in the HRS is used to discriminate pattern and noise, i.e., in particular, spike integration is enabled only for the presentation of an input pattern, to prevent a decay of the overall accuracy due to noise (Ambrogio et al., 2016b).

Figure 4B shows the measured weights of the 16 PCM synapses, divided in pattern synapses and background synapses which were not stimulated by input pattern spikes. Once the internal potential overcomes the threshold V_*TH*_, the POST generates a spike, thus enabling the synaptic potentiation/depression (depending on the PRE/POST spike delay) and the increase of the homeostatic PCM conductance. In turn, the PCM conductance increase causes the increase of *V*_*TH*_, hence the homeostatic control mechanism. This is evidenced by the decreased POST spiking frequency in Figure 4C, which ensures an improved energy efficiency of the SNN. The integration is disabled when the POST fires in order to avoid the integration of set/reset pulses to prevent excessive charge storage in the integrator block of Figure 2.

Figure 4D shows a simplified schematic to explain the management of the homeostatic neuron for the STDP measurements in a WTA network. An Arduino 2 (or Mega2560) microcontroller acts as master of the whole setup, managing both the gate voltages and the proper top electrode biases of the synaptic elements implemented with PCMs. The microcontroller also manages the results of the integration signal with respect to the adaptive internal thresholds of the homeostatic neurons. Note also that, at fire, the multiplexers enable the passage of the top electrode voltage of the synapses in order to implement the STDP learning paradigm.

### 3.1. Fashion-MNIST Accuracy and Robustness

**Figure 5:**
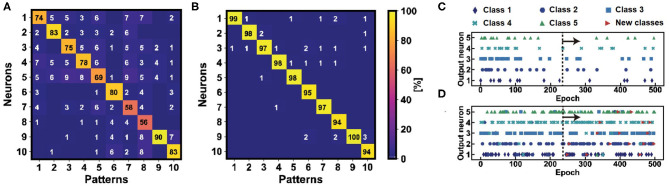
Confusion matrices for the study of the average accuracy of the learning activities for the 10 classes of the Fashion-MNIST training dataset without **(A)** and with **(B)** homeostasis. The learning accuracy highlights a high and stable tendency only when homeostatic neurons are used as post-synaptic neurons in the WTA network, reaching a value of 97%. **(C)** Fire activities of 5 homeostatic and non-homeostatic **(D)** neurons in 500 epochs of pattern and noise presentations. The homeostatic neurons are robust against “false” patterns presentations from another dataset, e.g., MNIST (here submitted after the 250*th* epoch).

To study the effect of homeostatic scaling on multi-pattern unsupervised learning, we simulated our SNN for the average classification of images from the Fashion-MNIST dataset, characterized by 10 different classes of clothes. Figure 5 shows the confusion matrices from Monte Carlo simulations for the learning accuracies without homeostasis (Figure 5A) and with homeostasis (Figure 5B). The study is carried out by considering one image for each of the 10 classes of the training dataset, replicating the study for the available 60,000 images and implementing the WTA protocol with a single-layer perceptron of 784 input neurons and 10 output neurons for each case (Ambrogio et al., 2016a). The learning accuracies are then averaged for each class to assess the overall efficiency. Homeostatic scaling allows for an accuracy increase by about 20% on average for the pattern specialization during learning of ten different images from the Fashion-MNIST dataset, which highlights the importance for unsupervised learning of PCM-based adaptive threshold. Such adaptive mechanism is also fundamental for achieving better accuracy in deep neural networks, where the homeostatic scaling improves the neuronal specialization for a pattern of a specific class of the dataset (Martin et al., 2020). The improvement of the accuracy can be directly referred to the better specialization achieved by the control PCM device which assures an optimized threshold level for each specific neuronal spiking activity. In fact, the homeostatic mechanism allows to exceed the threshold only when the learnt pattern appears at the input. Note that, thanks to the additional bitline of Figure 4A used for pattern/noise detection, the low-density inputs are neglected, thus avoiding spurious firing activity.

Homeostatic scaling also improves the robustness of the network for the classification when external perturbations, such as disturbs, errors or false patterns from other datasets, are presented at the input. To test the classification robustness of the network, Figure 5 show the output neuronal spikes during the classification of five images from Fashion-MNIST with homeostasis (Figure 5C) and without homeostasis (Figure 5D). In the first phase of the experiment, five images from Fashion-MNIST are presented and classified. In this phase, the non-homeostatic neurons show some errors due to the lack of a dedicated “specialization,” while no significant errors are evident among the homeostatic neurons. In the second phase of the experiment, handwritten digit patterns from the MNIST dataset are presented along with the Fashion-MNIST patterns. The homeostatic neurons do not show erroneous spikes since they have been specialized on the Fashion-MNIST patterns during the previous learning procedure. On the other hand, the non-homeostatic neurons show spurious spikes in correspondence of the presentations of the false patterns, due to the fact that the similarity between the patterns of the two datasets is sufficient to induce a false fire. Such behavior is avoided using the threshold modulation mechanism which allows to set a specific threshold for a specific learnt pattern, thus highlighting the higher classification robustness thanks to the homeostatic scaling procedure.

### 3.2. Active Forgetting by Conductance Drift

The PCM device is programmed by set pulses (with current I_*SET*_) and reset transitions. The variation of the resistive distributions can be modulated by incremental application of pulsed signals at the top electrode of the device, thus enabling multilevel states. These states are affected by conductance drift if the device is not constantly re-programmed in time. During standard STDP procedures, the conductance drift does not affect the overall behavior of the network, since the devices are continually set and reset in the pattern and background positions. Similarly, the internal state used to calibrate the threshold does not suffer too, since the drift effect is not appreciable in the reference timescale, as already seen in Figure 4C.

**Figure 6:**
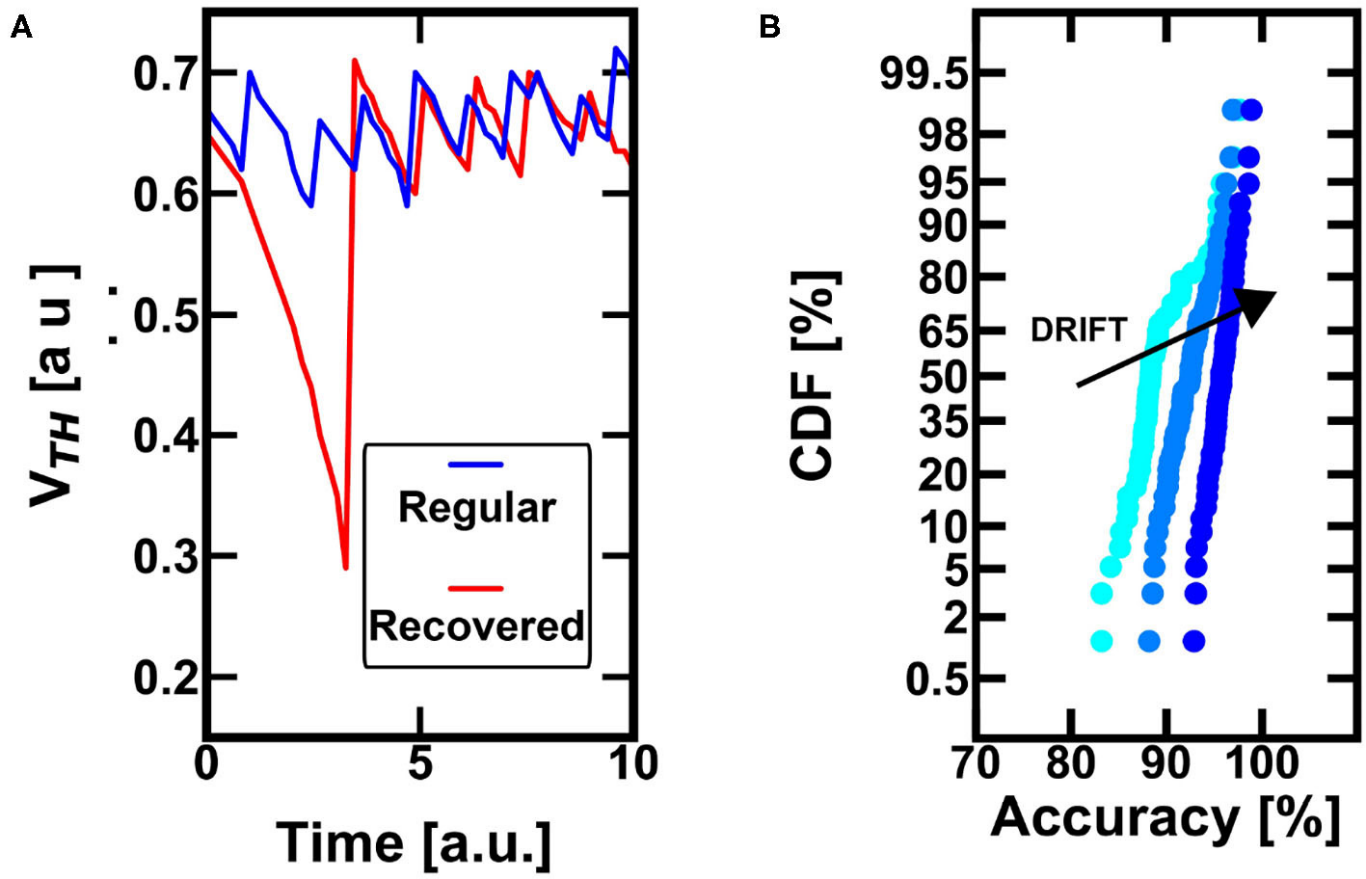
**(A)** The conductive drift leads to a substantial decrease of the threshold whenever the neuron is not excited (and the device is not reprogrammed), red line. This behavior well fits the bio-inspired forgetting and can lead to the recovery of a wrong spiking specialization toward improved classification. On the other hand, blue line, if the neuron is regularly excited (even if not often in time), the drift effect does not lead to active forgetting. **(B)** The conductance drift of the PCM devices has a positive effect for the recovery of neurons which committed error during the classification, such as neurons that have specialized on “wrong” patterns. At increasing drift of the control PCM device, the internal threshold gets progressively smaller, and the neuron is induced to fire again to the presentation of another pattern (eventually the “good” one). This favorable scenario is due to the fact the pattern information is correlated in time, while the errors are not. Thus, the drift effect can recover the error and increase the probability of accurate spiking activity in time.

STDP has been recently used in the final classification layer of deep convolutional networks for achieving continual learning (Muñoz-Martín et al., 2019). In this kind of neural networks, the convolutional filters generate responses which constitute artificial patterns that are learnt and classified afterwards via unsupervised WTA STDP. This procedure enables the incremental learning of new patterns during inference, since the convolutional filters give (for the new classes) a combination of responses which is original with respect to the others. However, since the variability among the new artificial patterns is high there is the possibility of having neurons which commit errors, specializing on input patterns that are unlikely to appear again at the input of the WTA STDP. In this situation, the internal PCM device is not activated for a long time, thus causing a decrease of the threshold, as shown by the Monte Carlo simulations in Figure 6A. Here, in particular, you can see that a regular spiking activity continually adjusts the threshold of the device, thus avoiding the lowering of the threshold. On the other hand, once a spurious spike activity is taken into consideration (red line), the internal threshold decreases considerably in time, since the spurious firing activity is not correlated. Note that such behavior can induce a neuron to change specialization, since the reduction of the threshold is proportional to an increase of neuronal excitability.

Furthermore, the conductance drift in time could be directly referred to the bio-plausible active forgetting, which erases previously stored information as a complementary procedure with respect to the homeostatic scaling consolidation (Davis and Zhong, 2017). Such active scaling forgetting gets rid of the unwanted pattern specialization and allows for a further specialization neuron able to be dedicated to more likely patterns at the input. Figure 6B shows the Monte Carlo simulations of the probability of recovering a past incorrect spiking event toward a fair accurate specialization at decreasing threshold conditions. In particular, it is evident that, increasing the conductance drift in time, it is possible to increase the firing excitability too. This is very relevant, since an incorrect specialization due to an uncorrelated error can be recovered by the correct excitation of a time-correlated input (i.e., a pattern), which is far more probable to contribute to the firing activity. Note that the presented figure is referred only to previously misunderstood firing activities, that are the only cases for which the drift plays a positive role.

## 4. Homeostatic Neuron in Recurrent Neural Networks

The bio-inspired spike-frequency adaptation modulates the fire excitability of a neuron inside a neural network. In other words, the fire responsivity directly depends on the past specialization history of the network. Such behavior along a temporal sequence is the key element for the recurrent neural networks (RNN) which can be thus re-designed taking advantage of the SFA mechanism (Amit, 1989).

**Figure 7:**
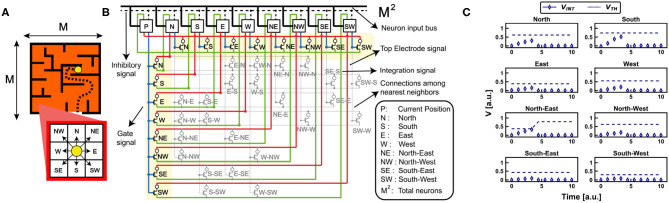
**(A)** Case study maze for the investigation of the reinforcement learning capabilities of the bio-inspired RNN. **(B)** The synapses of the RNN link symmetrically each neuron to and from the nearest neighbors. At every position P, the neuron sends a signal to the synaptic gates of its neighbors. When one neuron integrates enough current to overcome its internal threshold, it fires and inhibits all the network. Every firing activity maps the movement of the agent. The schematic of the circuit also shows the connections among the nearest neighbors. **(C)** Experimental measurement for a single movement of the agent inside the case study maze. The nearest neurons start to integrate current until one (North-East neuron) overcomes the threshold and fires. Note that the fire activity of the neuron causes an increase of the PCM internal‘threshold.

To support the spike-frequency adaptation of the neurons for reinforcement learning tasks, we considered a free-model decision-making test where an agent has to move in an environment until it finds a global reward. In particular, we considered the navigation problem of Figure 7A, where an agent explores the maze via penalties and rewards until it is successful in finding the escape path. In this case study, each point of the environment is configured as a homeostatic neuron which modulates its internal state as a function of the firing history of that particular position inside the environment. In particular, the reward is given when the agent reaches the prize causing the decrease of the internal threshold of the rewarded positions, while the punishment arises when the agent touches a barrier causing the increase of the internal threshold (Frémaux et al., 2013). Once the agent finds the escape path, it starts to remember the successful way by progressive rewards, i.e., the internal thresholds of successive positions decrease. Thus, the network evolves relying only on the self-adaptive threshold mechanism of reward and penalty and on the synaptic plasticity, without any further external aid.

We addressed the problem of a maze of size MxM (M = 30) by a brain-inspired RNN with M^2^ self-adaptive neurons, where each neuron represents a position within the maze. Figure 7B shows a section of the RNN limited to the current position P and the eight nearest neighbors, which map the eight fundamental cardinal directions. Note that the RNN is completely symmetrical, since each connection between the current position and one of the adjacent is configured by two symmetric synapses to and from P. Each synapse has a 1T1R structure where the PCM device is randomly initialized in HRS or LRS. Note that the further synapses connecting the nearest neurons also contribute to the definition of a symmetric matrix with respect to the diagonal of the RNN. Synaptic weights along the diagonal are all zero because a neuron, i.e., a position, is not self-connected. Note that an inhibitory signal enables a WTA algorithm, as already described in the first section of this manuscript.

### 4.1. The Movement of the Agent

The environmental boundaries are initially defined by programming the thresholds of each position. The goal of the network is to find the escape route across the maze via reinforcement learning, thus supporting the relevance of the PCM plastic properties for typical neuromorphic abilities (Frémaux et al., 2013).

At any time, only the occupied neuron P is activated by external spike stimulation. The firing activity of the neuron P induces two types of event: first, the threshold V_*TH*_ of neuron P increases, due to the homeostatic mechanism; second, nearest neighbor neurons are stimulated by the spiking activity of neuron P. This dynamics was experimentally validated by the RNN with PCM neurons and synapses of Figure 7B, where each neuron is connected to the nearest neighbor positions, e.g., E is connected to P, NE and SE. Figure 7C shows the measured internal potential V_*INT*_ for the eight nearest neurons during stimulation of neuron P with an external spiking signal of limited duration. Since all synapses are initially programmed in random state (i.e., 50% in LRS, 50% in HRS), only those neurons which are connected by synapses with relatively high conductance show substantial current integration. Once the first neuron reaches the threshold, namely neuron North-East in the example of Figure 7C, the agent moves to the corresponding position and a new cycle can be started by zeroing the internal potential V_*INT*_ of all the neurons (i.e., the typical inhibitory signal already discussed for the WTA network). Note that, as the agent position changes, the synaptic weights must be reinitialized to enable trial-to-trial variations of the random walk, thus boosting the effect of penalties and rewards. Note also that the self-adaptive threshold mechanism induces partial crystallization of the control PCM of the firing neuron, thus preventing the agent to come back to previously occupied positions. In fact, as visible in Figure 7C, once a neuron fires it increases its internal threshold, thus making less probable the coming back to that position from the surrounding ones during the next movements of the agent.

### 4.2. Penalty/Reward Mechanisms and Optimization of the Solution

**Figure 8:**
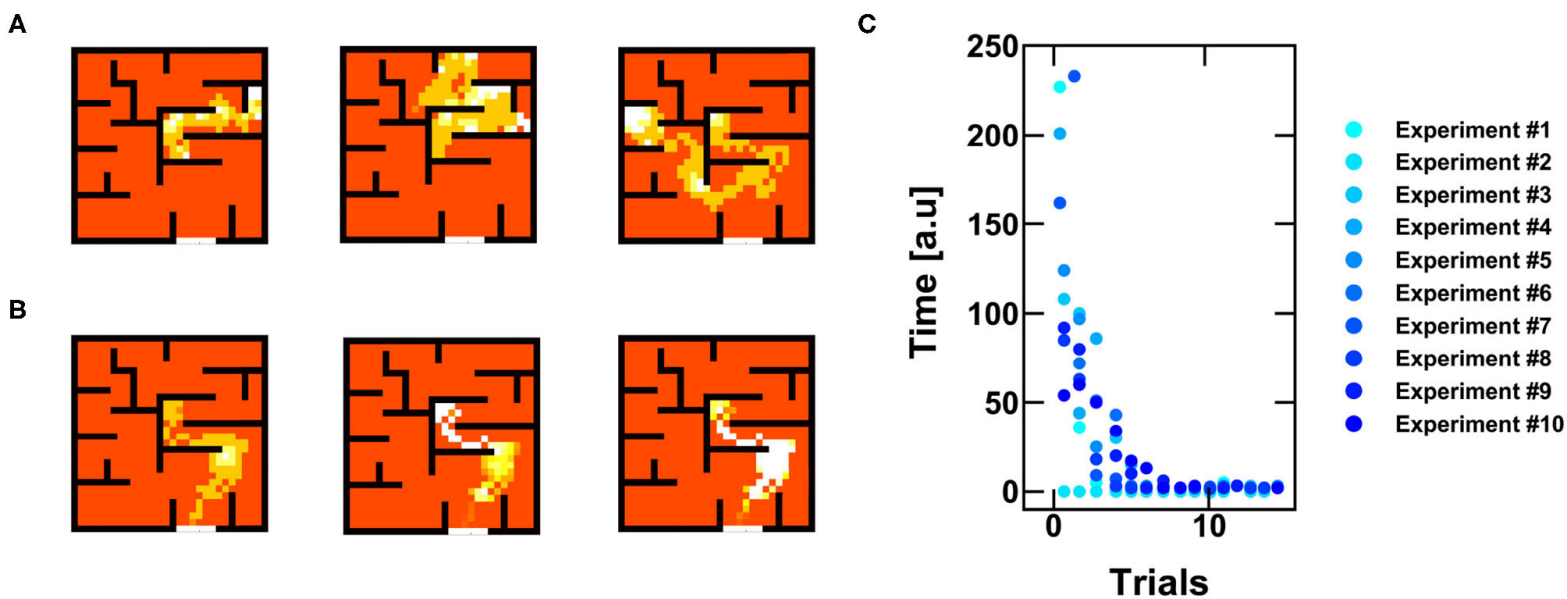
**(A)** Example of three random walks for successive trials of exploration of the agent. **(B)** Example of three successive trials after finding the escape path: the agent progressively improves its policy for finding the reward, eventually not committing errors anymore. **(C)** Time to get to the escape path as a function of the number of trials for 10 different experiments of 15 trials each.

Figure 8A shows the random walks of the agent during successive trials. Each experiment is limited in time, since the agent has to find the reward by elaborating a strategy, rather than testing each single position (Frémaux et al., 2013). If the agent cannot escape within 400 spikes, (i.e., steps of the agent), a new trial starts by reinitializing the agent position and the synaptic weights. The reinforcement learning is instead retained from trial to trial and only relies on (i) penalties, when the agent touches a wall, or (ii) rewards, when the escape paths is found. Both penalties and rewards are mapped by acting on the internal *V*_*TH*_ of the neuron, thus increasing or decreasing the neuronal responsivity. When the agent touches a wall, a penalty is assigned to that position by increasing the corresponding *V*_*TH*_. On the other hand, when the agent finds the escape path, a reward is given by lowering the *V*_*TH*_ of the last positions occupied by the agent.

As shown in Figure 8B, once the escape path has been disclosed, the system tends to follow the preferential path toward the objective. This happens because the reward policy introduces a positive feedback, which reduces the *V*_*TH*_ of the path thus improving the preference of the agent to follow the escape path. Figure 8C shows the time to find the reward as a function of successive trials. Note that the reward has two main effects, namely (i), the system self-optimizes its policy map by increasing the time efficiency, and (ii) the spiking activities concentrate in the positions close to the target, thus reducing any unwanted energy consumption along ordinary positions which do not give any reward. As a result, the experience-based evolution of our RNN relies on PCM-based neurons and synaptic plasticity and enables the optimization of reinforcement learning for autonomous decision-making navigation.

### 4.3. Impact of Drift on Reinforcement Learning

**Figure 9:**
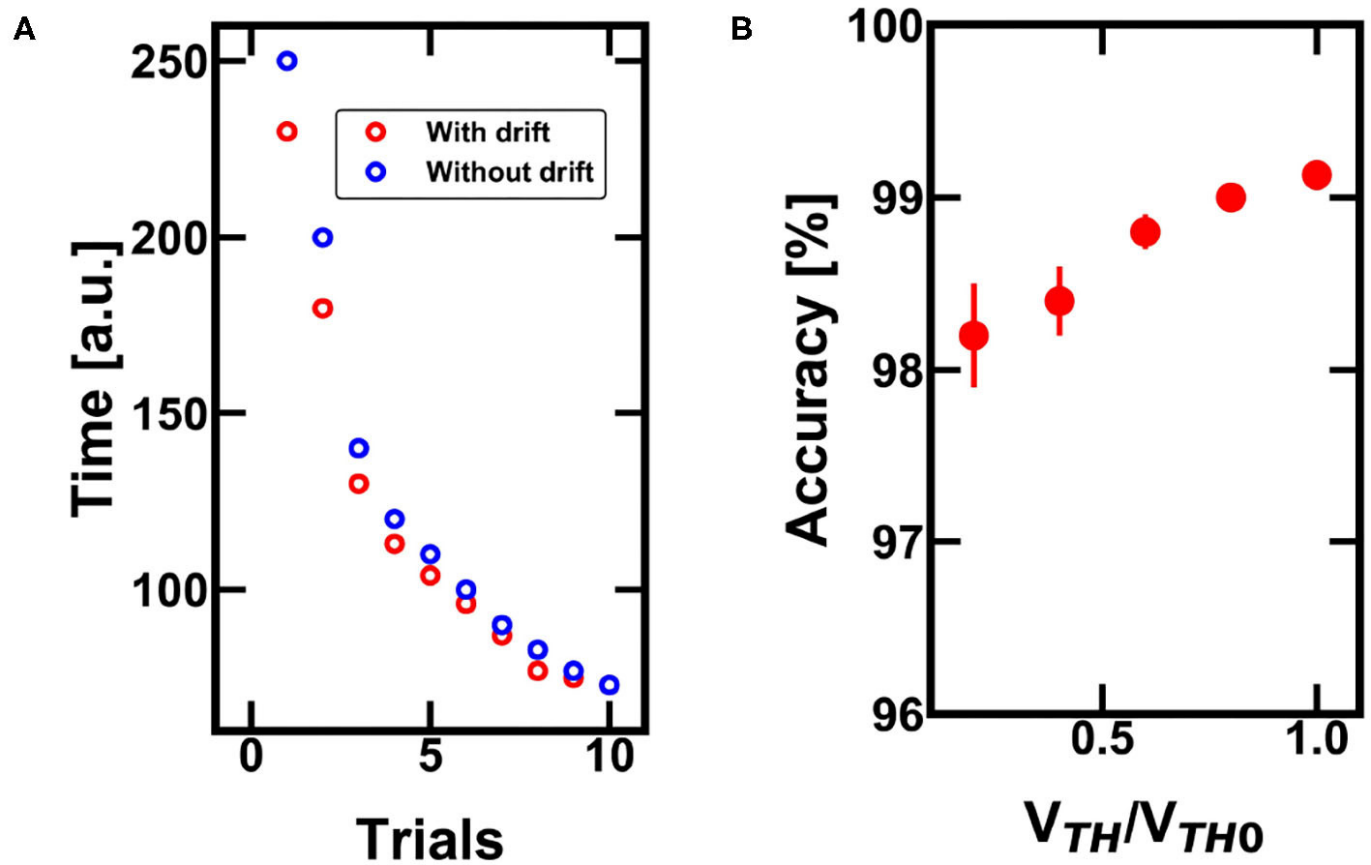
**(A)** Monte Carlo simulations of the minimum time needed to successfully find the escape path with and without the drift effect of the PCM devices. The larger the drift, the lower the time to get to the final reward. **(B)** Impact of the drift on the accuracy for finding the escape path over 1,000 trials of the same experiment. Note that the drift is not a benefit since the decreasing V_*TH*_ (with respect to the nominal *V*_*TH*0_) can lead to misunderstanding in the policy map definition.

To study the impact of the drift, we studied the effect of the drift-induced decrease of the internal neuronal threshold in Figure 9A. The decrease of the internal threshold causes a decrease of the necessary time to get to the final reward for each trial. On the other hand, the drift also affects the threshold of the punished neurons, but the drift does not drive such positions to a condition comparable with the ordinary ones.

The difference between the reinforcement learning with and without PCM drift decreases at increasing trial of specialization, since the reward facilitate the identification of the successful path by acting on the threshold of the corresponding positions (less integration time per single step is needed to follow the rewarded path). Figure 9B shows the accuracy (i.e., the ability of finding the escape path considering a fixed number of trials per experiment) over 1,000 Monte Carlo simulations. The study indicates that the drift of the PCMs increases the error probability, lowering the overall efficiency of the network. As a result, drift does not introduce significant benefit in the case of reinforcement learning, with respect to the STDP learning. In more complex situations, where the surrounding boundaries change continuously thus requiring a constant reconfiguration by the agent, the drift-induced forgetting mechanism could become favorable, since it would boost the quest toward other points of the environment.

### 4.4. Energetic Efficiency

**Figure 10:**
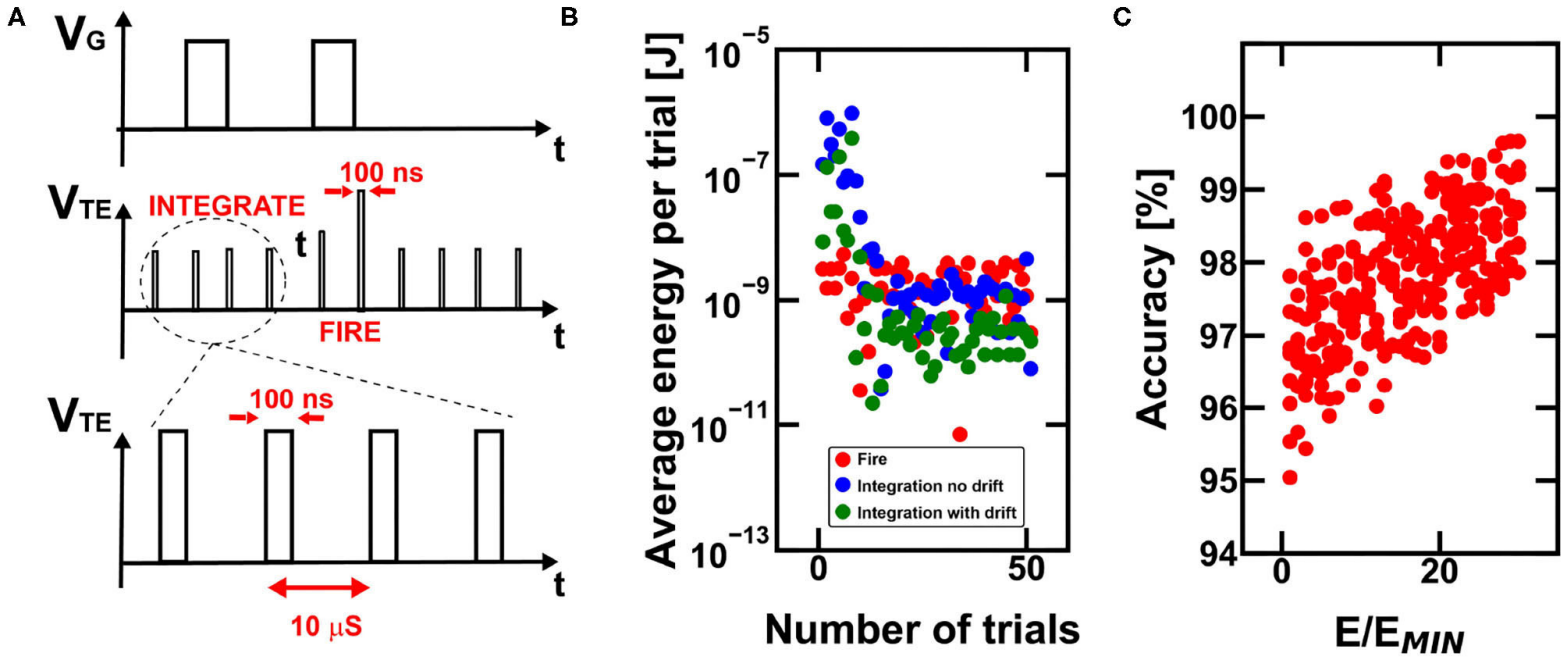
**(A)** Burst-mode operation for power-saving during PCM-based working procedure of the RNN. **(B)** Note that the required energy for the operations carried out by the RNN is dependent on the grade of specialization of the network and on the final achievement with respect to the disclosure of the escape path. In fact, once the final reward is found, the network progressively decreases the total need of integration energy. Note that the simulated energy consumption reduction also comes with a decrease in the overall accuracy for finding the escape path when conductance drift is considered. **(C)** Monte Carlo simulations of the global accuracy for the case study maze considering increasing trial and error procedures for the programming of the internal state and of the inter-neuronal synaptic devices.

The energy efficiency of reinforcement learning can be improved by operating the devices in burst-mode (Bianchi et al., 2019), which consists of the application of fast pulsed signals at the electrodes of the PCM devices, thus enabling a consistent reduction of the required energy per single operation. In our simulations, we stimulated the devices with pulsed signal with duration of 100 ns separated by silent periods of 10 μs as shown in Figure 10A.

Figure 10B shows the average energy per single exploration trial of the agent, indicating that the energy consumption decreases as the agent refines its strategy. During the initial trials, the energy consumption due to integration needed to explore the environment is larger than the other contributions, since the agent requires many steps to explore the surroundings. Once the final reward is achieved, the integration procedure requires less energy, thanks to the threshold decrease in the path positions close to the objective. Note also that the simulation without drift indicates a higher integration energy, which is due to the fact that the internal states undergo a decrease of the respective threshold due to conductance drift, thus requiring less power per single trial. The energy consumption decrease, as well as the time decrease to get to the solution, depends on the timescale of the reinforcement learning execution in hardware, since longer times means larger conductance drift.

Figure 10C shows the accuracy for finding the reward as a function of the number of memory access per single device (e.g., the PCM internal state of the neurons) in order to assure the theoretical conductance value assessed during the simulations. However, a 30 times higher energy consumption for best programming condition only improves the accuracy by 1.5%, on average. This result indicates the substantial robustness and efficiency of bio-inspired neuromorphic computing for reinforcement learning tasks.

## 5. Continual Learning in Artificial Neural Networks

**Figure 11:**
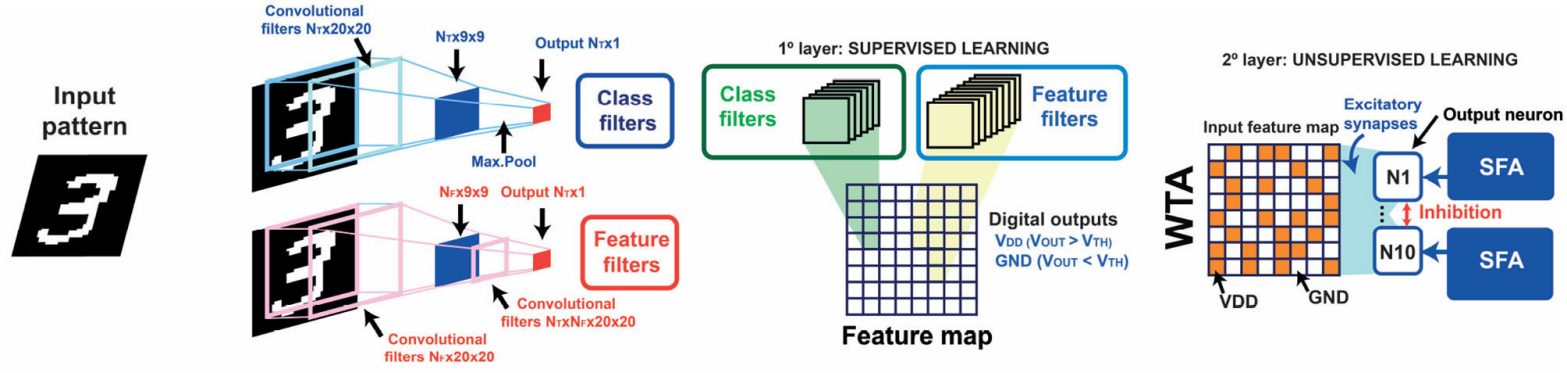
Schematic architecture of the hybrid supervised-unsupervised neural network. The input patterns coming from the dataset are convolved with pre-trained convolutional filters. Each filter, which can recognize a generic feature, “feature-filter,” or a specific class, “class filter,” gives a single-bit response (found/not found response). The responses of the convolutional filters give thus rise to a binary feature map, which is then classified by homeostatic neurons using the STDP paradigm in the WTA architecture.

STDP-based unsupervised learning with homeostatic neurons is a robust approach for achieving continual learning in artificial neural networks. In particular, STDP has been already introduced in the last layer of convolutional neural networks (CNNs) in order to get resilience in neural systems trained with the backpropagation algorithm (Muñoz-Martín et al., 2019). These kinds of hybrid supervised/unsupervised neural networks rely on custom training algorithms to extract, after convolution, single-bit responses per each filter relative to a found/not found trained feature, as illustrated in Figure 11. After convolution, a novel feature map arises, which is then classified by means of post-synaptic neurons under the STDP learning paradigm. In order to study the effect of the introduction of PCM-based SFA neurons in this neural system, we built a WTA network with ten POSTs capable of spike frequency adaptation, as in Figure 2, and inhibitory signals. The inhibition, in particular, enables the drop of the internal potential of all the neurons when a fire event occurs (Pedretti et al., 2017; Bianchi et al., 2020c).

**Figure 12:**
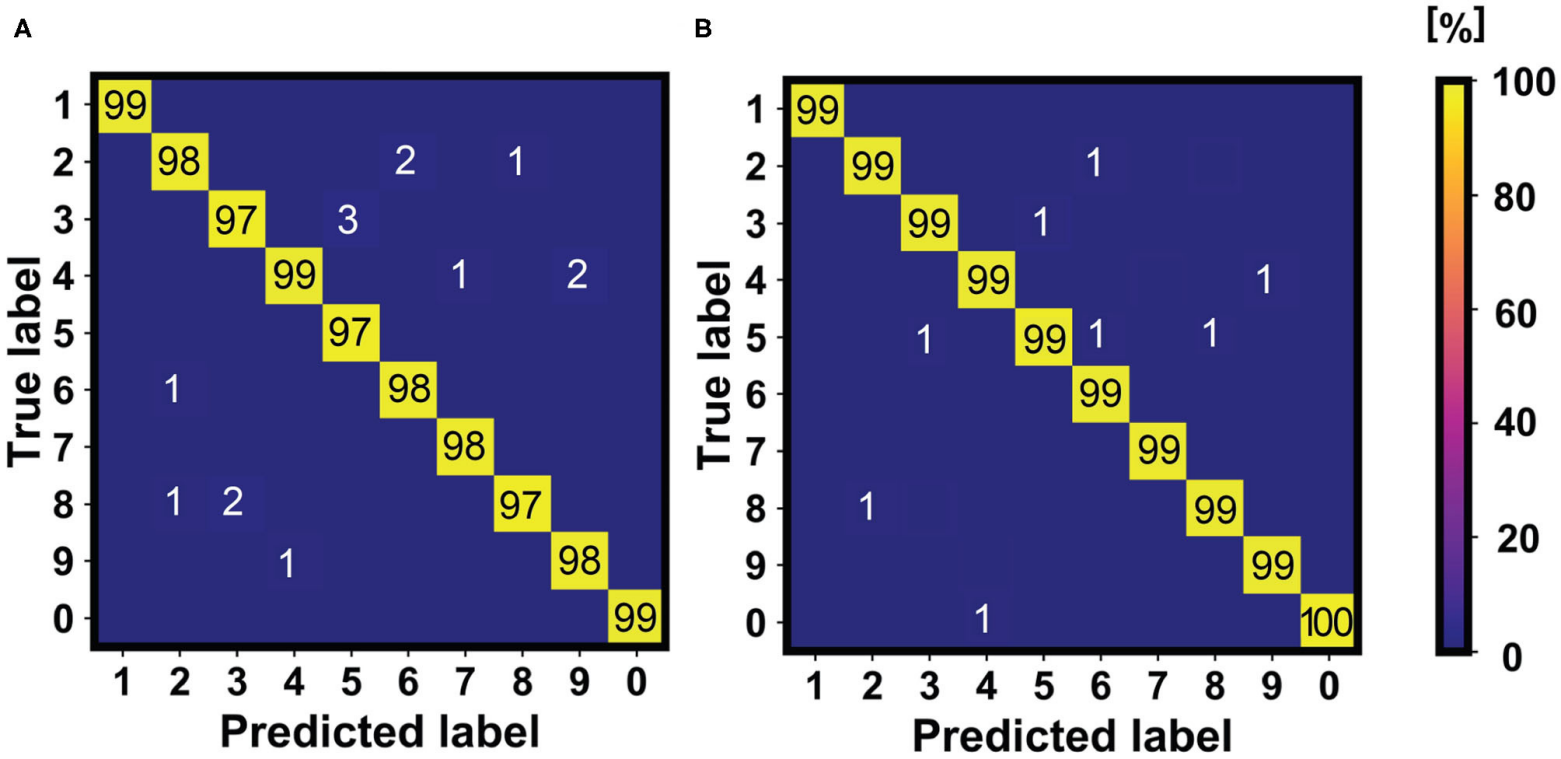
Comparison of the accuracies of previous works (Muñoz-Martín et al., 2019) **(A)** with the accuracies obtainable after using homeostatic neurons in the last layer of the network **(B)**. Note that the accuracy results increase in the second case, which is mainly due to the improved specialization capability and to the active forgetting mechanism introduced by the SFA homeostatic neurons.

The use of neurons with SFA control mechanism in the last layer of the network of Figure 11 introduces robustness and improved accuracy with respect to previous works, as reported in Figure 12 for the inference of the MNIST dataset (10,000 patterns of handwritten digits). This is due to two main contributions, namely: (i) the improved specialization capability of the neurons to get specialized on specific input patterns (each neuron modulates its internal threshold on a specific feature map arising from the patterns joining the same class, as also studied in Figure 5); (ii) errors in the WTA classification are prone to be corrected thanks to the spontaneous forgetting mechanism studied in Figure 6. This latter point, in particular, is due to the fact the classification errors are not correlated in time, thus driving a wrong fire event to be forgotten in time.

Thus, the homeostatic neurons appear as key elements to introduce both resilience and accuracy in artificial neural networks, paving the way for the next technological steps of artificial intelligent computation.

## 6. Conclusions

In this work we introduced a novel artificial neuron based on phase change memory (PCM) devices capable of internal regulation via homeostatic and plastic procedures. The neuron relies on the definition of the internal threshold by multilevel programming of the control PCM devices, thus enabling the specialization of large patterns and the continual learning capability of CNNs by introducing the STDP procedure in a supervised framework. The novel neuron is also used to introduce a bio-inspired recurrent neural network which directly creates a directed experienced-graph in time by keeping trace of the fire history of each neuron of the network. Such recurrent connections based on neurons capable of spike frequency adaptation demonstrate decision-making capabilities for navigation tasks. Furthermore, we show that conductance drift of the PCM devices can be used to emulate active forgetting in neural networks. This work supports the suitability of PCM devices for the optimization of synaptic dynamics and the implementation of brain-inspired computing in artificial intelligence.

## Data Availability Statement

The data that support the findings of this study are available from the corresponding author upon reasonable request.

## Author Contributions

IM-M and SB have contributed equally in the planning, design and implementation of the system, the extraction and the interpretation of the results, the figures realization, and the text writing. SH, GP, and OM have contributed to the experimental setup. DI has supervised the planning and the design of this project. All authors contributed to the article and approved the submitted version.

## Conflict of Interest

The authors declare that the research was conducted in the absence of any commercial or financial relationships that could be construed as a potential conflict of interest.

## Publisher's Note

All claims expressed in this article are solely those of the authors and do not necessarily represent those of their affiliated organizations, or those of the publisher, the editors and the reviewers. Any product that may be evaluated in this article, or claim that may be made by its manufacturer, is not guaranteed or endorsed by the publisher.
